# A History of Cord Blood Banking and Transplantation

**DOI:** 10.1002/sctm.17-0075

**Published:** 2017-04-29

**Authors:** Joanne Kurtzberg

My interest in blood stem cells and cord blood dates back to my fellowship in Pediatric Hematology/Oncology at Duke. During my fellowship, I worked on the development of novel antileukemia drugs based on analogs of purine metabolism and treated a teenage patient with refractory T‐cell acute lymphoblastic leukemia with a novel anti‐leukemia drug, 2′‐deoxycoformycin, an inhibiter of adenosine deaminase. During 5‐day course of therapy, his leukemia converted from a T‐lymphoid to a myeloid phenotype before our eyes 1. I subsequently established a cell line from these leukemic cells, DU‐528 and, ultimately, proved that this leukemia arose from a common lymphoid‐myeloid progenitor cell 2, which then led me to the study of hematopoietic stem and progenitor cells. Hal Broxmeyer, who would go on to become a pioneer in the use of cord blood as a source of donor cells for bone marrow reconstitution, mentored me as I studied normal and malignant hematopoietic stem cells isolated from bone marrow, fetal liver, and umbilical cord blood. In the clinic, I cared for children with leukemias and blood dyscrasias, including a young boy named Matthew Farrow from Salisbury, NC, with Fanconi anemia (FA) and evolving bone marrow failure. This genetic disease, which arises from a mutation in genes that encode the enzymes responsible for DNA repair, is associated with a host of serious medical and developmental problems. The prognosis for Matthew's condition was stark: most children with Fanconi anemia died of bone marrow failure or leukemia in the first decade of life, unless treated with a bone marrow transplant from a human leukocyte antigen (HLA)‐matched donor.

Matthew did not have a living related matched donor, but when his mother became pregnant with another baby, the unborn fetus was tested to see if she was affected with FA and, if not, was a match for Matthew. An amniocentesis was performed and samples were sent to Arlynn Auerbach, a renowned researcher at the Rockefeller University in New York who was the first to describe the mutations responsible for Fanconi anemia and who established the FA patient registry 3. Dr. Auerbach confirmed that Matthew's sibling was healthy and an HLA identical match. What happened next was a remarkable convergence of insight, dedication, and cooperative innovation in this emerging therapeutic area.

At that time, the only potentially curative therapy for Fanconi anemia was a bone marrow transplant from a healthy matched related donor. However, at the Memorial Sloan Kettering Cancer Center (MSKCC), a team led by Ted Boyse, Judy Bard, and Hal Broxmeyer was exploring the potential uses of cord blood in transplantation and cell therapy. Although cord blood hitherto was routinely discarded as medical waste, the trio had formed a company, Biocyte, to develop therapeutic applications for cord blood and obtained a patent for the freezing and banking of cord blood for future use. Dr. Broxmeyer's research had confirmed that cord blood was enriched for highly proliferative hematopoietic cell progenitors—even more so than bone marrow—and hypothesized that cord blood could serve as a substitute donor for bone marrow transplantation 4. Meanwhile, Dr. Boyse had performed preliminary testing of the concept in mice, although biological differences limited the usefulness of animal‐model testing,

Dr. Auerbach, who had been working with Broxmeyer on some of the biological aspects of patients with Fanconi anemia who converted from one disease phenotype to another, was aware of a mutual interest. The group had been hoping to pilot their therapeutic approach in human patients, but their plans had recently been put on hold when a family interested in participating turned out not to be candidates for the procedure after all—in their case, the potential donor sibling was found to also have Fanconi anemia. It was at this point that Dr. Auerbach approached us to ask if we might be interested in using Matthew's sister's cord blood as the donor for his transplant. We talked with Matthew's parents, who talked with Matthew, then a young 5 years of age, to determine whether he thought the transplant was a good idea. It was Matthew who ultimately made the final decision, which was yes.

Gordon Douglas, an obstetrician from New York Hospital traveled to and stayed in Salisbury, NC, for several weeks to attend the delivery of Matthew's sister so that he could collect her cord blood. Shortly after the birth of Matthew's sister, the cord blood was collected into a wide mouthed bottle containing heparin to prevent clotting. HLA typing was confirmed at Duke, and the cord blood was taken to Hal Broxmeyer's laboratory at MSKCC and cryopreserved, after addition of dimethyl sulfoxide but without other processing, in his research liquid nitrogen freezer. We asked Dr. Eliane Gluckman, an expert in transplantation of patients with FA at the L'Hopital St. Louis in Paris, if she would perform the transplant, and she agreed. We waited until Matthew's baby sister was 6 months old so that she could serve as a back‐up bone marrow donor if the cord blood cells failed to engraft. Through an international, industry, and academic collaboration, monies were raised to send Matthew and his family to Paris for the transplant. Blue Cross Blue Shield and L'Hopital St. Louis covered the medical expenses. Biocyte supported the couriering by Dr. Broxmeyer of the cord blood unit from New York to Paris. Matthew was prepared for transplantation with attenuated doses of chemotherapy and total lymphoid irradiation because, due to the defect in DNA repair, patients with FA cannot tolerate the usual myeloablative doses of chemotherapy and radiation. Dr. Broxmeyer hand‐carried the frozen sample in a dry shipper—one seat on the plane for him, and one for the cord blood cells—arriving the day of the transplant. Although Matthew experienced an infusion reaction, there were no significant complications and 19 days later, he engrafted with his baby sister's cord blood cells, effectively curing Matthew of Fanconi anemia 5. Now in his early thirties, he is leading a healthy, normal life.

Following this early success, other researchers extended the work by treating other HLA‐matched siblings using cord blood, usually for leukemia 6. Cord blood not only worked, but had advantages over bone marrow transplantation, including lower rates of acute graft‐versus‐host disease, a complication of transplantation in which the donor cells attack the recipient. This finding was of particular interest to researcher Pablo Rubinstein, at the New York Blood Center, who was investigating whether unrelated banked cord blood could be used successfully in recipients lacking a match in their family or in the unrelated adult donor registry. With the support of a research grant from the National Heart, Lung, and Blood Institute (NHLBI), Pablo established the first public cord blood bank in the U.S. In 1993, my team at Duke, which had opened our pediatric transplant program in 1990, performed the first unrelated donor cord blood transplant in a 4‐year‐old boy with T‐cell leukemia. Over the next 2 years, 25 transplants were performed, and the success of these first transplants established cord blood as a viable alternative donor for patients unable to identify a matched related or unrelated donor for transplantation and paved the way for the entire field 7. Subsequent reports from the New York Blood Center's bank confirmed these results in children 8 and adults 9.

Over the next few years, cord blood was utilized as an alternative donor of hematopoietic stem and progenitor cells for hematopoietic stem cell transplantation (HSCT) in all of the indications that previously utilized bone marrow donors. This included treatment of patients with hematological malignancies, congenital immunodeficiency syndromes, bone marrow failure, hemoglobinopathies, and inherited metabolic diseases 8, 9, 10. The NHLBI sponsored a program called “COBLT” from 1997–2004, which funded the establishment of three additional public cord blood banks 11 and five prospective, multicenter studies testing the use of cord blood donors in children with hematological malignancies 12 and children with nonmalignant genetic diseases 13. While initially challenged by the fact that a typical cord blood unit lacked sufficient cells to reliably engraft in an adult‐sized individual, in 2005, Juliet Barker and John Wagner showed that using two cord blood units for one transplant resulted in improved rates of engraftment and survival in adults 14. From 2005–2012, I was a co‐principal investigator with Dr. John Wagner on a multicenter, randomized trial to test whether two cords would be superior to one cord blood graft in children with hematologic malignancies 15, but surprisingly, in children who could receive an adequate cell dose from a single cord blood unit, one cord was sufficient.

Fast‐forwarding to the present day, banked unrelated donor cord blood is a routine source of donor cells for patients lacking a matched related or unrelated adult donor. Nearly 800,000 unrelated units are banked in public banks, and more than 5 million samples are stored in private banks. In 1998, Duke established the Carolinas Cord Blood Bank under the COBLT program, and today it is one of the largest banks in the U.S. Today, banked unrelated donor cord blood is the only hematopoietic stem cell source to be regulated by the U.S. Food and Drug Administration, and to date, seven public cord blood banks in the U.S. have been granted Biologic License Applications.

In addition to the use of banked unrelated donor cord blood as a source of donor cells for HSCT, cord blood is now emerging as a promising new cell therapy. Cord blood is not just a “bag of blood stem cells,” and newer therapies are leveraging the activities of monocytes and other cells in the mix. To this end, we have manufactured a macrophage/microglial‐like cell from cord blood to utilize as an adjunctive intrathecal therapy in children with inherited leukodystrophies undergoing HSCT 16. This cell promotes remyelination of the brain in animal models and may arrest disease progression more rapidly in these patients 17. In addition, the safety of autologous cord blood infusions in children with cerebral palsy and other acquired brain injuries, without preparative chemotherapy or immunotherapy, was demonstrated by me and one of my junior colleagues at Duke in 2010 18. Subsequently, we are testing cord blood as a cell therapy working through paracrine signaling, which promote endogenous cellular repair in patients with hypoxic ischemic encephalopathy 19, adult stroke, and as shown in the paper published in the inaugural cord blood section of this journal, autism [20]. While results are preliminary, they create hope for cord blood‐derived cellular therapies as novel treatments for diseases that cause life‐long disabilities and currently have no curative options.

In my opinion, cord blood is 18 years young and still has a long developmental pathway ahead. It is remarkable that a routinely discarded substance can save lives. While the potential of cord blood in HSCT is largely realized, it will continue to expand access to donors for patients of minority ancestries who need donors for HSCT for malignant and nonmalignant diseases, such as sickle cell anemia. More excitingly, I predict that the use of cord blood cells, in both autologous and allogeneic settings, as cellular therapies in the emerging field of regenerative medicine, currently in its infancy, will emerge as one of the major great advances in novel therapeutics in medicine over the next decade.
